# Periodontal disease of secondhand smoking patients

**DOI:** 10.1186/1617-9625-12-S1-A11

**Published:** 2014-06-06

**Authors:** Arzu Beklen, Deniz Uckan, Gulten Tsaous Memet

**Affiliations:** 1 Department of Periodontology, Faculty of Dentistry, Osmangazi University, Eskisehir, 26480, Turkey; 2 Institute of Biomedical Engineering, Bogazici University, Istanbul, 34684, Turkey; 3 Medico-social Centre, Dental Clinic, Bogazici University, Istanbul, 34684, Turkey; 4 Tepebasi Oral and Dental Health Hospital, Ankara, 06400, Turkey

## Background

Our cross-sectional study investigated associations of cumulative exposure to secondhand smoke and duration of smoking cessation with periodontitis among family members. Furthermore the effects of other risk factors on these associations were analyzed as well.

## Materials and methods

In total 109 patients were analyzed with their full mouth periodontal examination. The groups were divided as current second hand smokers, former second hand smokers and non-smokers. The associations between periodontitis occurrence and potential risk factors was analyzed using univariate and multivariate analysis.

## Results

The results showed that the rates of periodontitis among nonsmokers, former second hand smokers, and current second hand smokers were 19.5%, 22.3%, 37.4% respectively. We then adjusted the other periodontal risk factors and we found that the odds ratio (95% confidence interval) for periodontitis was 2.12 for former second-hand smokers and 3.56 for current second hand smokers.

## Conclusions

In summary, not only a significant dose-response relationship between pack-years of second hand smoking and periodontitis presence was observed, but also a significant decrease in the occurrence of periodontitis was observed in second hand smoke stopped group.

